# New findings of shallow water caprellids (Crustacea, Amphipoda, Caprellidae) from Uruguay with an illustrated key to species

**DOI:** 10.3897/zookeys.1279.175887

**Published:** 2026-05-11

**Authors:** Taiara Ramos, Ana Verdi, José M. Guerra-García

**Affiliations:** 1 Sección Entomologìa, Facultad de Ciencias, Universidad de la República, Igúa 4225 CP11400, Montevideo, Uruguay Facultad de Ciencias, Universidad de la República Montevideo Uruguay; 2 Laboratorio de Biología Marina, Departamento de Zoología, Facultad de Biología, Universidad de Sevilla, Avda Reina Mercedes 6, 41012 Seville, Spain Facultad de Biología, Universidad de Sevilla Seville Spain

**Keywords:** *
Caprella
*, new records, *
Paracaprella
*, Southwestern Atlantic, taxonomy

## Abstract

There is a lack of studies focusing on caprellid amphipods along the coasts of Uruguay. So far, only three species, *Caprella bathytatos*, *C. equilibra*, and *C. penantis*, have been recorded from Uruguay. Sampling was carried out for one year (from April 2022 to March 2023) along the Uruguayan coast between latitudes 34°02'43.4"S and 34°55'05.7"S at rocky outcrops in the localities of Piriápolis, Punta Ballena, La Barra, La Paloma, La Pedrera, and Punta del Diablo. At each sampling point, scraping was performed in intertidal and shallow waters up to a depth of 10 m by snorkeling and SCUBA. All substrata, consisting mainly of algae and bryozoans, were collected within a 50 × 50 cm grid. Additionally, in Punta del Diablo, buoyant material stranded on the beach was also collected. This work represents the first record of the species *C. andreae*, *C. dilatata*, *C. scaura*, and Paracaprella aff. pusilla for Uruguay, increasing the number of known species in the country to seven. Herein, an illustrated key to the seven species known to occur in Uruguay is presented. The low caprellid diversity in Uruguay could be related to the highly variable abiotic characteristics of the Uruguayan coast, mainly due to the freshwater input from the Río de la Plata, and to other biotic factors such as the low diversity of macroalgae. The taxonomical impediment (lack of taxonomists and scarce sampling in the area) could also cause an underestimation of species richness. Therefore, additional sampling efforts, especially in deeper waters and sediment habitats, are still necessary to properly characterize this amphipod group in Uruguay.

## Introduction

The family Caprellidae Leach, 1814 includes 464 species classified into three subfamilies, Caprellinae Leach, 1814, Paracercopinae Vassilenko, 1972, and Phtisicinae Vassilenko, 1968 (Horton et al. 2025). They inhabit marine ecosystems and can constitute one of the dominant taxa in intertidal and shallow water communities. Most of the caprellids live as epibionts on a wide variety of natural substrata, including algae, seagrasses, sediments and marine invertebrates such as hydrozoans, bryozoans, sponges, ascidians, echinoderms, and crustaceans (McCain 1968; Laubitz 1970; Takeuchi and Hino 1997; Serejo 1998; Guerra-García 2001). Caprellids are characterized by a reduced abdomen, a head fused with the first thoracic segment, and a tendency to reduce or lose the third and fourth pair of pereopods (Caine 1974). Pleopods used by other amphipod families for swimming are reduced or absent in caprellids, resulting in virtually no swimming ability (Caine 1978). This, together with the lack of a planktonic larval stage, suggests that cosmopolitan species have the ability to disperse by clinging to floating material (Takeuchi and Sawamoto 1998). In fact, the broad distribution of some species could be explained by their frequent association with fouling communities on natural or artificial substrates, including floating objects (Thiel and Gutow 2005; Ros et al. 2013a).

Caprellids feed on suspended materials, prey on other organisms, or graze on epibiotic fauna and flora (Caine 1974) and most of them can be considered as detritivores (Guerra-García and Tierno de Figueroa 2009). Recent research has demonstrated that caprellids can be useful bioindicators of marine pollution and environmental stress (e.g., Guerra‐García and García‐Gómez 2001; Takeuchi et al. 2001; Guerra-García et al. 2010a; Navarro-Barranco et al. 2020) and are potential resource for aquaculture (Woods 2009; Baeza-Rojano et al. 2010, 2014).

There is an increasing attempt to improve the knowledge of caprellids along the coasts of South American, including studies conducted in Colombia (Guerra-García et al. 2006a), Venezuela (Díaz et al. 2005), Brazil (Wakabara et al. 1991; Wakabara and Serejo 1998; Mauro and Serejo 2015; Cunha et al. 2018; Serejo and Siqueira 2018), Argentina (Lopez Gappa et al. 2006; Chiesa and Alonso 2014), Chile (Guerra-García and Thiel 2001; Thiel et al. 2003; Beermann et al. 2025), Peru (Chunga-Llauce et al. 2022, 2023a, 2023b) and Ecuador (Soler-Hurtado and Guerra-García 2016). Regarding Uruguay, there is a lack of information on caprellid amphipods. Indeed, only three species have been reported along Uruguayan coast so far, *Caprella penantis* Leach, 1814, *C. equilibra* Say, 1818, and *C. bathytatos* Martin & Pettit, 1998 (Wakabara et al. 1982; Scarabino 2006; Verdi and Celentano 2008), reflecting the scarce effort made to date in taxonomic studies on caprellids. Indeed, most of the caprellids cited in Uruguay are records at the family level, without determination of the species, as part of general survey studies of communities associated with rocky substrata (Riestra et al. 1992; Brazeiro et al. 2006; Demicheli 2017; Ramos 2017). Given the critical role played by caprellids within marine ecosystems and the deficit of taxonomic information, this study aims to shed light on the knowledge about the family Caprellidae along the Uruguayan coast.

## Materials and methods

**Figure 1.**
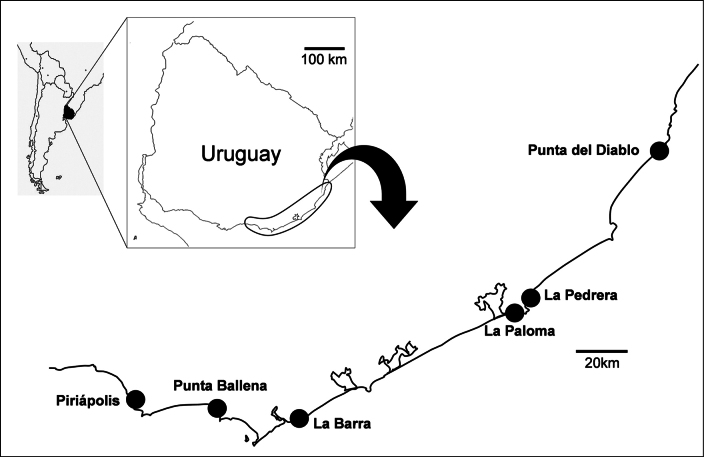
Study area showing the sampling localities along the Uruguayan coast.

Sampling was carried out during one year (from April 2022 to March 2023) along the Uruguayan coast between latitudes 34°02'43.4"S and 34°55'05.7"S at rocky outcrops in the localities of Piriápolis (34°53'01.65"S, 55°16'48.6"W), Punta Ballena (34°54'35.5"S, 55°02'45.6"W), La Barra (34°55'05.7"S, 54°51'13.7"W), La Paloma (34°39'49.9"S, 54°10'10.7"W), La Pedrera (34°35'24.4"S, 54°07'16.3"W), and Punta del Diablo (34°02'43.7"S, 53°32'0.8"W) (Fig. 1). At each sampling point, scraping was performed in intertidal and shallow waters (up to 10 m deep by snorkeling and SCUBA) to collect all substrata (mainly algae and bryozoans) within a 50 × 50 cm grid. Additionally, in Punta del Diablo, buoyant material stranded on the beach (mainly consisting of plastic litter and seaweeds) was also collected, sieved, and fixed in 70°alcohol for preservation. In the laboratory, the samples were sorted and caprellids were identified. Lateral view figures of each species were drawn using a Leica compound microscope equipped with a camera lucida and final images were produced following Takeuchi (2015) methodology. Classification follows Lowry and Myers (2013).

All the examined material is deposited in the scientific collections of Entomology Section, Laboratory of the Faculty of Sciences, Montevideo, Uruguay (**CCSEFC**) except for a male and female specimens of each species (used for lateral view figures), which were deposited in the Museo Nacional de Ciencias Naturales, Madrid, Spain (**MNCN**).

## Results

A total of 463 individuals were examined, identifying six species belonging to two genera. Four of these six species are new records for the Uruguayan coast.

### Systematics

#### Superfamily Caprelloidea Leach, 1814

**Family Caprellidae Leach, 1814**

**Subfamily Caprellinae Leach, 1814**

##### Caprella andreae

Taxon classificationAnimaliaAmphipodaCaprellidae

Mayer, 1890

26D60C19-118E-5526-B804-3B8289DEB9EC

Fig. 2

Caprella acutifrons f. Andreae Mayer, 1890: 51–55, pl. 2, fig. 38; pl. 4, figs 56, 70, 71.—Chevreux and Fage 1925: 452, fig. 430A.
Caprella andreae McCain, 1968: 19, figs 8, 9, 55.—Krapp-Schickel 1993: 777, fig. 530.—Aoki and Kikuchi 1995: 54–61, figs 1, 2.—Cabezas et al. 2013a: 483–497, fig. 1.—Sezgin et al. 2009: 433–437.—Woods et al. 2014: 97–102, figs 2–4.

###### Material examined.

Uruguay • 13 males, 14 females; Punta del Diablo; 34°02'43.7"S, 53°32'0.8"W; buoyant material stranded on the beach; 16 March 2023; Ramos T.; CCSEFC 342. • 1 male, 1 female; same collection data as for preceding; MNCN 20.04/20991.

**Figure 2.**
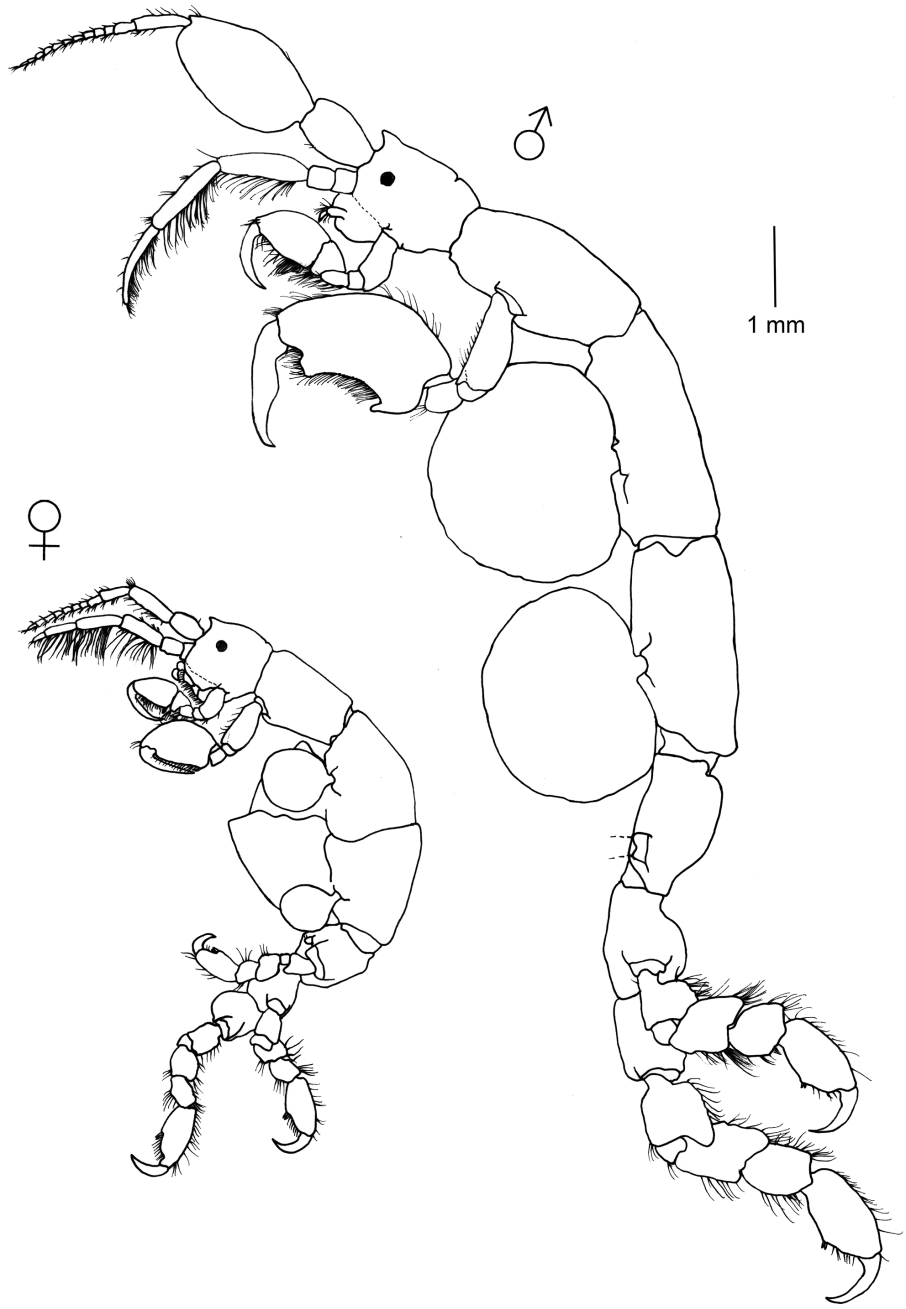
*Caprella andreae* Mayer, 1890. Lateral view of male and female collected on the beach of Punta del Diablo (floating material), 16 March 2023 (34°02'43.7"S, 53°32'0.8"W), Uruguay (MNCN 20.04/20991).

###### Remarks.

Male specimens of *C. andreae* from Uruguay agree with original and subsequent descriptions of the species, having antenna 1 with globose peduncular article 2, gills large and rounded and propodus palm of pereopods 5–7 convex (Mayer 1890; McCain 1968; Krapp-Schickel 1993). *Caprella andreae* mainly differs from other related congeneric species in the convexity of the propodus of pereopods 5–7, which is probably an adaptation to cling to floating objects or tiny algae on the turtle carapace (Aoki and Kikuchi 1995; Cabezas et al. 2013a). Although more than 25 caprellid species have been found on floating objects and have been reported or inferred as facultative rafters (see Thiel and Gutow 2005; Ashton 2006), only *C. andreae* can be considered as an obligate rafter, living exclusively on rafts where it spends its entire cycle (Cabezas et al. 2013a). This species has been collected from the carapaces of sea turtles, floating objects such as driftwood, pumice, floating seaweeds, buoys, or ropes (McCain 1968; Cabezas et al. 2013a; Sezgin et al. 2009; Woods et al. 2014; Hoffman 2022). The known distribution of *C. andreae* includes Northeastern Atlantic, Mediterranean Sea, Hawaii, Sea of Japan, Korean Strait, Atlantic coast of USA, Cuba, and Brazil (Woods et al. 2014; Mauro and Serejo 2015). The present study provides the first record of the species from the coasts of Uruguay, representing the southernmost record of the species in the Atlantic. Woods et al. (2014) also found the species in a mussel farm in New Zealand, where it seems to be successfully established. Considering that there is no information on *C. andreae* populations other than on floating objects or vagile fauna, further research is necessary to compare populations on floating material with populations on fixed structures such as the mussel farm (Woods et al. 2014). Phylogenetic analyses conducted by Cabezas et al. (2013a) revealed the potential existence of cryptic species within *C. andreae* populations, so the species requires further taxonomic resolution.

##### Caprella dilatata

Taxon classificationAnimaliaAmphipodaCaprellidae

Krøyer, 1843

75A76FE2-8C65-54EB-A00B-66718EC4B99B

Fig. 3

Caprella dilatata Krøyer, 1843, 585–590, pl. 8 figs 1–9.—McCain 1968: 38.—Krapp-Schickel 1993: 779, fig. 532.—Guerra-García et al. 2006b: 100–108, fig. 2E, G.—Masunari and Takeuchi 2006: 49–60, figs 1–5.—Cabezas et al. 2013b: 85–99, fig. 5g.
Caprella acutifrons f. *minor* Mayer, 1890: 54, pl. 2, fig. 35; pl. 4, figs 54, 64.
Caprella acutifrons f. *typica* Mayer, 1890: 54, pl. 2, fig. 34; pl. 4, figs 62–63.

###### Material examined.

Uruguay • 12 males, 15 females; La Paloma; 34°39'49.9"S, 54°10'10.7"W; intertidal, on red filamentous algae; 1 September 2022; • 12 males, 53 females; Punta del Diablo; 34°02'43.7"S, 53°32'0.8"W; 2–4 m deep, on red filamentous algae; 16 March 2023; • 8 males, 10 females; intertidal, on red filamentous algae; La Pedrera; 34°35'24.4"S, 54°07'16.3"W; 15 March 2023; Ramos T.; CCSEFC 340. • 1 male, 1 female; Punta del Diablo; 34°02'43.7"S, 53°32'0.8"W; 2–4 m deep, on red filamentous algae; 16 March 2023; Ramos T.; MNCN 20.04/20992.

**Figure 3.**
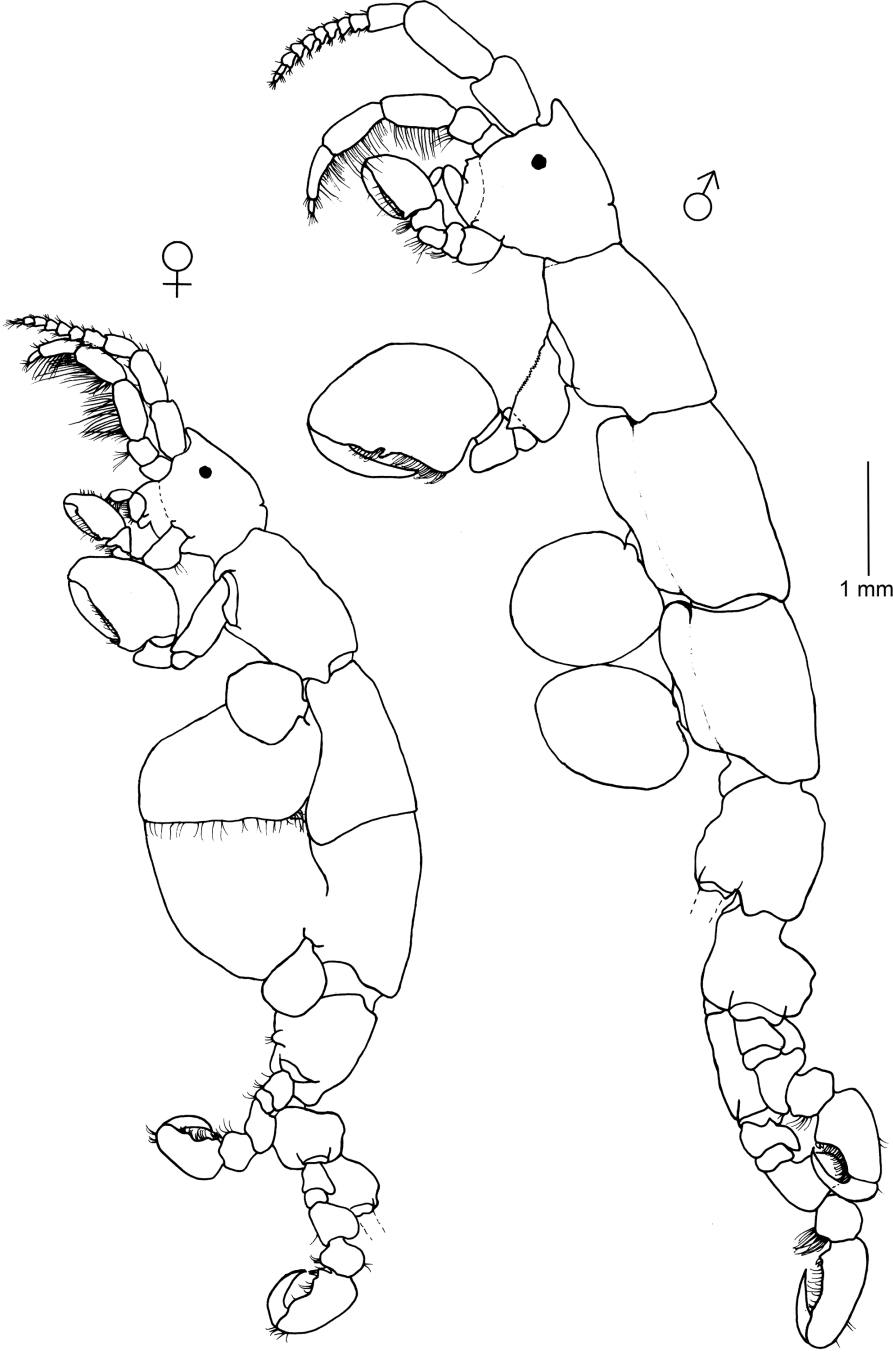
*Caprella dilatata* Kroyer, 1843. Lateral view of male and female collected from red filamentous algae, rocky outcrops (2–4 m deep), 16 March 2023, Punta del Diablo (34°02'43.7"S, 53°32'0.8"W), Uruguay (MNCN 20.04/20992).

###### Remarks.

*Caprella dilatata* was originally described by Krøyer (1843) based on material from Rio de Janeiro, Brazil and it has been recently redescribed in detail by Masunari and Takeuchi (2006), based on Brazilian material from Itapocoroi Bay, Santa Catarina state. The present distribution of the species includes the Atlantic Ocean and the Mediterranean Sea (McCain and Steinberg 1970). Masunari and Takeuchi (2006) reported the occurrence of *C. dilatata* in Argentina, and the present study represents the first record of the species for Uruguay, confirming the wide distribution of the species along the Atlantic coast of South America. The material from Uruguay agrees with the descriptions of Mediterranean and Atlantic specimens (see Krøyer 1843; Krapp-Schickel 1993; Masunari and Takeuchi 2006). *Caprella dilatata* is morphologically very close to *C. andreae* and *C. penantis*, being the three species distributed in Uruguay. The most relevant distinguishing characters are the gills (rounded in *C. andreae* and *C. dilatata* and elongate in *C. penantis*) and the propodus palm of pereopods 5–7 (convex in *C. andreae* and concave in *C. dilatata* and *C. penantis*). Furthermore, the morphology of gnathopod 2 also differs among the three species (see Guerra-García et al. 2006: figs 2, 3; Cabezas et al. 2013b: fig. 5). All the material collected from Uruguay has been found clinging onto red filamentous algae from intertidal and very shallow waters. The species has also been found in moderately exposed hard bottom dominated by brown seaweeds (Jacobucci et al. 2002). Lacerda and Masunari (2011) reported the presence of *C. dilatata* in 11 different substrata along the Brazilian coast, being a dominant species in finely branched and softly surfaced algal substrata, ascidian and bryozoan colonies, and the surfaces of buoys and ropes. Based on substratum selection experiments, these authors pointed out the strong preference of *C. dilatata* for the original alga-substratum and highlighted the importance of camouflage by keeping similar coloration to the substratum. In Itapocoroi Bay, *C. dilatata* reaches high densities over the surface of mussel shells, attached to longlines of mussel farming, and in associated bryozoans and sponges or directly on the surface of floats or ropes that hold longlines (Masunari and Takeuchi 2006). These authors pointed out that they become so numerous in the summer months that they might cause irritations to farmers’ skin. Important populations of *C. dilatata* have also been found in some recreational marinas of southern Spain, associated to fouling communities (mainly bryozoans and hydroids) on floating pontoons, and feeding mainly on detritus and secondarily on crustaceans, hydroids, microalgae, and dinoflagellates (Guerra-García et al. 2015). Nuñez-Velazquez et al. (2017) studied the population dynamics of *C. dilatata* inhabiting Mar del Plata Harbour, Argentina, and found that the species was present during the whole year, reaching its maximum densities in May, with no correlation between seawater temperature and monthly density. Baeza-Rojano and Guerra-García (2013) studied the *C. dilatata* life history under laboratory conditions (20 °C, salinity 35, 12 hours light/12 hours dark) and found that its life span from emergence of juveniles to death was 28–71 days, the generation period was 30.4 days, reaching sexual maturity in 21–32 days and having a fast incubation time from 3–5 days. Molting processes were continuous along the entire life cycle, with a number of 4–11 molts.

##### Caprella equilibra

Taxon classificationAnimaliaAmphipodaCaprellidae

Say, 1818

70C4D5A1-1DF3-5EF8-9740-699BD1C9DBCC

Fig. 4

Caprella equilibra Say 1818: 391–392.—McCain 1968: 25, figs 12–13.—Krapp-Schickel 1993: 782–783, fig. 533.—Guerra-García and Thiel 2001: 878–879, fig. 6.—Díaz et al. 2005: 4, fig. 4.
Caprella aequilibra Mayer 1882: 45, pl. 1, fig. 7; pl. 2, fig. 1–11; pl. 4, figs 20–25; pl. 5, figs 16–18.—Chevreux and Fage 1925: 455, fig. 433.^1^

###### Material examined.

Uruguay • 24 males, 37 females; Piriápolis; 34°53'01.65"S, 55°16'48.6"W; intertidal, on green filamentous algae; 20 April 2022; Ramos T.; CCSEFC 389. • 1 male, 1 female; same collection data as for preceding; MNCN 20.04/20993.

**Figure 4.**
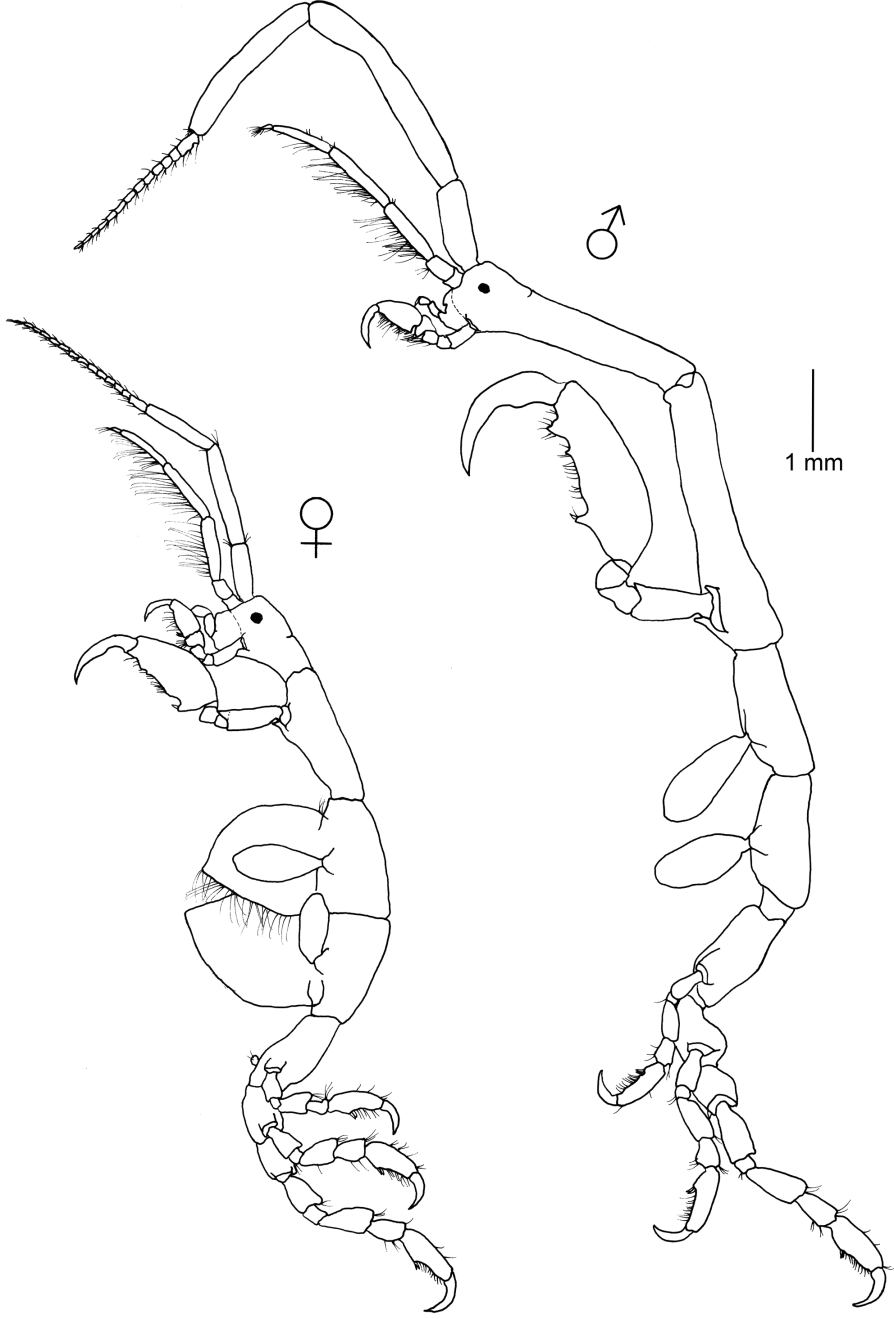
*Caprella equilibra* Say, 1818. Lateral view of male and female collected from green filamentous alga, rocky intertidal, 20 April 2022, Piriápolis (34°53'01.65"S, 55°16'48.6"W), Uruguay (MNCN 20.04/20993).

###### Remarks.

*Caprella equilibra* can be distinguished from other species of *Caprella* from Uruguay by the presence of a ventral projection between the insertions of gnathopod 2. This projection was present in most males and females, but it was lacking in some specimens. McCain (1968) also reported a variant of *C. equilibra* occurring along the coast of Virginia, North Carolina, and South Carolina in which the projection was reduced or absent. He pointed out that this reduction could be related to the association with gorgonians. However, in the present study, the specimens without projection were sharing the same habitat (green filamentous algae) as those with the projection. Zeina et al. (2015) found differences in robustness and size of specimens of *C. equilibra* from Madeira and Azores, and Guerra-García et al. (2015) also reported differences in size of specimens belonging to different populations of marinas from Southern Spain. The cosmopolitan distribution of *C. equilibra* could be explained by its ability to disperse in the water column as part of the plankton community (Takeuchi and Sawamoto 1998; Fernandez-Gonzalez et al. 2014; Guerra-García, pers. obs.) and to effectively colonize artificial structures (Ros et al. 2015; Revanales et al. 2022). However, considering the morphological variation among populations, a molecular approach would help to clarify its taxonomical status and to explore the existence or not of cryptic speciation (Sánchez-Moyano et al. 2015; Zeina et al. 2015). *Caprella equilibra* has been found on seagrasses, algae, sponges, hydroids, bryozoans, colonial ascidians, alcyonarians, and mussels, from the intertidal to 3000 m depth (McCain 1968; Krapp-Schickel 1993; Díaz et al. 2005). As the species is prone to colonize artificial environments (Ros et al. 2016), it can be abundant in aquaculture facilities, water duct pipes, power plants, as well as on floating substrata, e.g., buoys and floating algae (Takeuchi and Sawamoto 1998; Thiel et al. 2003; Fernandez-Gonzalez and Sanchez-Jerez 2017). *Caprella equilibra* feeds principally by filtering, frequently using grooming behavior (Guerra-García et al. 2002), and its diet mainly consists of detritus, crustaceans (e.g., copepods), hydroids, and dinoflagellates (Guerra-García and Tierno de Figueroa 2009; Alarcón-Ortega et al. 2012; Guerra-García et al. 2015). This species clings to the substratum mainly in the up-right position (Guerra-García et al. 2002). *Caprella equilibra* densities are related with seawater temperature (Nuñez-Velazquez et al. 2017). According to Baeza-Rojano and Guerra-García (2013) based on experiments conducted in laboratory conditions (20 °C, salinity 35, 12 hours light/12 hours dark) the caprellid life span for this species was 18–65 days, the generation period was 34.3 days, reaching sexual maturity in 23–33 days and having a fast incubation time from 4–6 days, and a number of 4–10 molts. Recently, the species has been considered a promising resource in aquaculture, as novel food with adequate nutritional profile rich in polyunsaturated fatty acids (Baeza-Rojano et al. 2014) and as an effective biofilter in Integrative Multi-Trophic Aquaculture (IMTA) systems (Guerra-García et al. 2016).

##### Caprella aff. penantis

Taxon classificationAnimaliaAmphipodaCaprellidae

667CA21F-D6B2-5351-95A9-414B1D95254D

Fig. 5

###### Material examined.

Uruguay • 63 females; La Paloma; 34°39'49.9"S, 54°10'10.7"W; 2–4 m deep, on red algae; 15 March 2023. • 1 male, 24 females; La Pedrera; 34°35'24.4"S, 54°07'16.3"W; intertidal, on red algae; 15 March 2023. • 7 males, 51 females; La Paloma; 34°39'49.9"S, 54°10'10.7"W; intertidal, on red algae; 7 June 2022. • 18 males, 64 females; La Barra; 34°55'05.7"S, 54°51'13.7"W; 2–4 m deep, on red algae; 6 December 2023. • 4 males, 4 females; Punta Ballena; 34°54'35.5"S, 55°02'45.6"W; 10 m deep, on red algae and bryozoans; 20 February 2023; Ramos T.; CCSEFC 388. • 1 male, 1 female; Punta Ballena; 34°54'35.5"S, 55°02'45.6"W; 10 m deep, on red algae and bryozoans; 20 February 2023; Ramos T. MNCN 20.04/20994.

**Figure 5.**
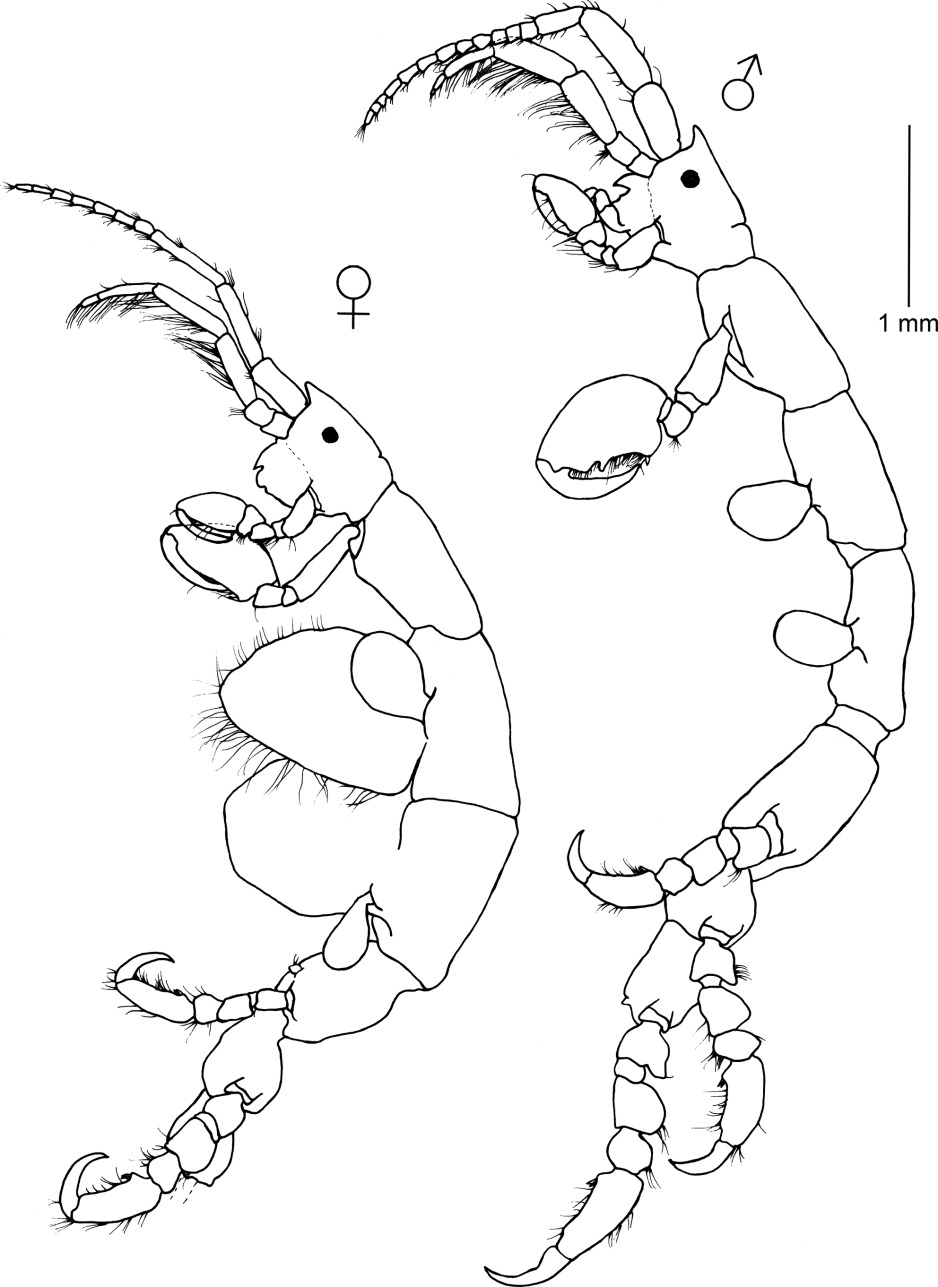
Caprella aff. penantis. Lateral view of male and female collected from red algae and bryozoans, rocky outcrops (10 m deep), 20 February 2023, Punta Ballena (34°54'35.5"S, 55°02'45.6"W), Uruguay (MNCN 20.04/20994).

###### Remarks.

*Caprella penantis* was considered one of the most taxonomically challenging caprellids in the world, having been recorded under several forms or subspecies names (Mayer 1890, 1903). Recently, hidden diversity and cryptic speciation have refuted cosmopolitan distribution in *C. penantis* (Cabezas et al. 2013b). Consequently, the study of this species must be conducted separately in each region since material traditionally assigned to *C. penantis* sensu lato can belong to undescribed species (e.g., Sánchez-Moyano et al. 2015). Furthermore, a recent molecular study (Cabezas et al. 2022) provided evidence that *C. penantis* sensu stricto is probably restricted to UK coasts (type locality), the northern coast of the Iberian Peninsula, and the Azores. Therefore, although some morphological characters of the specimens from Uruguay are in agreement with the material of *C. penantis* sensu stricto, Uruguayan specimens probably belong to a different species. *Caprella penantis* has also been recorded in nearby regions of Brazil and Argentina (Mauro and Serejo 2015; López Gappa and Sueiro 2007). Future molecular studies are necessary to review the *C. penantis* complex in the area and explore whether all the populations from the east coast of South America belong to the same species or not, and, consequently, describe them following an integrative approach.

##### Caprella scaura

Taxon classificationAnimaliaAmphipodaCaprellidae

Templeton, 1836

EC32C488-C683-5FBD-89E4-30AC1DC0C599

Fig. 6

Caprella scaura Templeton 1836: 191–192, pl. 20, fig. 6.—Bate 1862: 355, pl. 56, fig. 4.—Mayer 1882: 65.—Mayer 1890: 70–74, pl. 4, figs 40–51, pl. 6, fig. 41, pl. 7, figs 2, 35, 36.—Mayer 1903: 117–120 pl. 5, figs 13–18, pl. 10, fig. 11.—McCain 1968: 40–44, figs 17–18.—Guerra-García 2003a: 4–5, fig. 2.—Krapp et al. 2006: 1–18, figs 1–12.—Ito et al. 2010: 183–190.—Guerra-García et al. 2011: 2617–2622, fig. 1.—Ros et al. 2014a: 145–155, fig. 3.—Cabezas et al. 2014: 2221–2245.
Caprella nodosa Templeton, 1836: 191–192, pl. 21, fig. 7.
Caprella cornuta Dana, 1853: 816–817.
Caprella attenuata Dana, 1853: 817–819.^2^

###### Material examined.

Uruguay • 1 male, 1 female; Piriápolis; 34°53'01.65"S, 55°16'48.6"W; 2–4 m deep, on red filamentous and red algae; 7 March 2023; Ramos T.; CCSEFC 341. • 1 male, 1 female; same collection data as for preceding; MNCN 20.04/20995.

**Figure 6.**
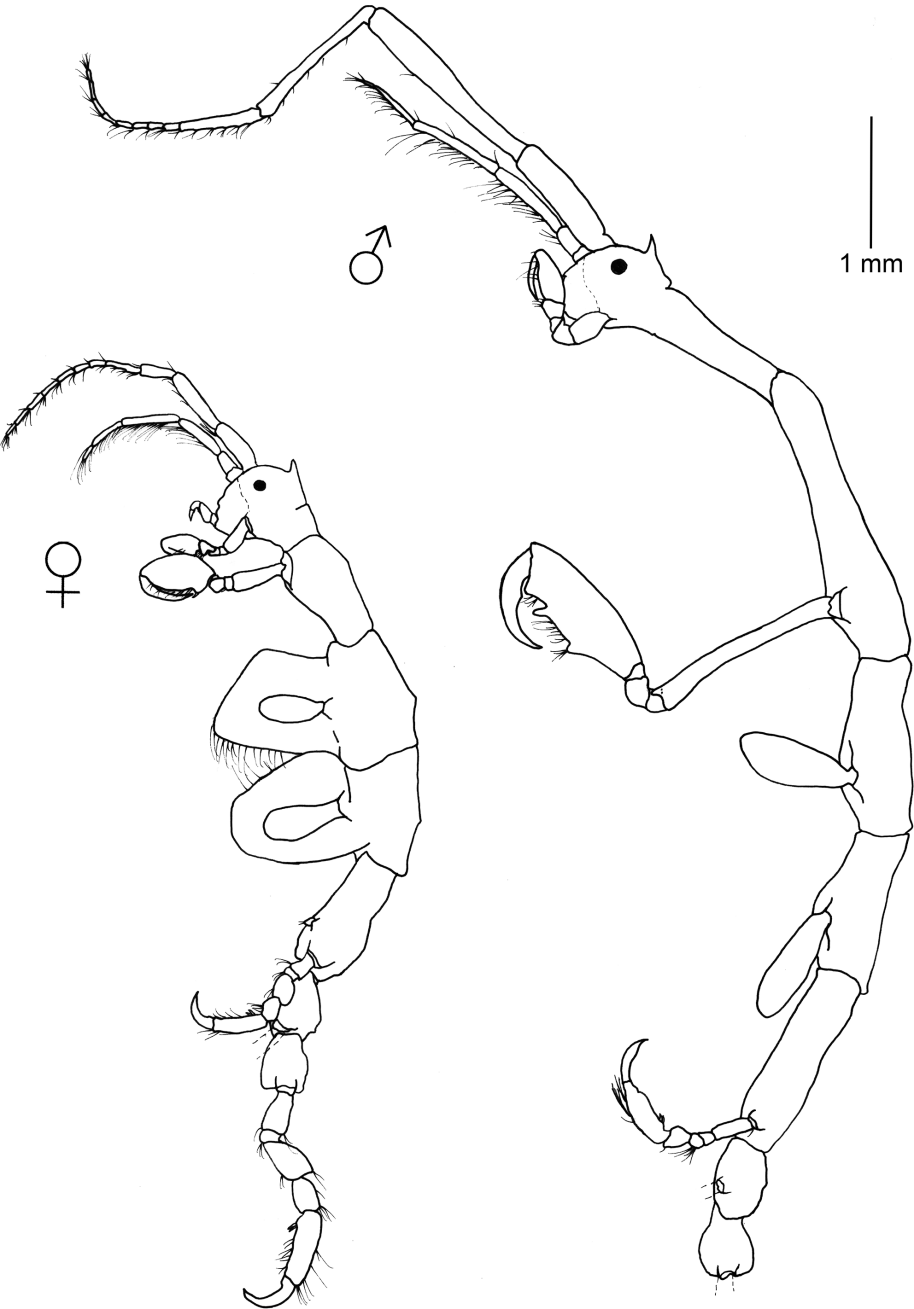
*Caprella scaura* Templeton, 1836. Lateral view of male and female collected from red filamentous and red algae, rocky outcrops (2–4 m deep), 7 March 2023, Piriápolis (34°53'01.65"S, 55°16'48.6"W), Uruguay (MNCN 20.04/20995).

###### Remarks.

*Caprella scaura* was described from Mauritius (the type locality) by Templeton in 1836. Mayer (1890, 1903) described six forms of the species: *typica* from Brazil and Australia, *diceros* from Japan, cornuta from Brazil, *californica* from California, scauroides from Hong Kong, China, and Japan, and *spinirostris* from Chile. Utinomi (1947) added a seventh form, *hamata* from Japan. The nominal species originally described from Mauritius is named *Caprella scaura scaura* (Krapp et al. 2006) and, according to morphological and molecular approaches (Cabezas et al. 2014; Ros et al. 2014a), *C. scaura scaura* and *C. scaura typica* are the same species, considered as *Caprella scaura* sensu stricto. *Caprella scaura californica* and *C. scaura scauroides* are now considered to belong to two separate species (Takeuchi and Oyamada 2013), and Cabezas et al. (2014) considered the subspecies *C. s. spinirostris* and *C. s. diceros* also as two valid species based on molecular evidence. The specimens from Uruguay totally agree morphologically with *Caprella scaura* sensu stricto, which is the only species within the complex with worldwide distribution due to its invasive character (Ros et al. 2014a; Martínez-Laiz et al. 2021). Similarly to Brazil, the species could be considered cryptogenic in Uruguay. In potentially native regions, this species occurs in natural habitats, inhabiting a variety of substrata such as bryozoans, hydroids, seaweeds, seagrasses, and sponges (Lim and Alexander 1986; Takeuchi and Hino 1997; Serejo 1998; Guerra-García 2003a). In the exotic range, the species is restricted to artificial environments, mainly recreational marinas, where it occurs mainly on bryozoans and hydroids (Ros et al. 2013a; Guerra-García et al. 2024). This species clings to the substratum mainly in the up-right position, although males spend higher percentages of time than females in the posture parallel to the substratum (González-Romero et al. 2016). It is mainly a detritivorous species (Guerra-García and Tierno de Figueroa 2009), using often grooming behavior (Guerra-García et al. 2002; Ros et al. 2014b). Maturation time has been estimated under laboratory conditions to require approximately 25 days (González-Romero et al. 2016). Abundance patterns, seasonal fluctuations, small scale distribution, and ecophysiology of *C. scaura* have been studied in localities along the Mediterranean and East Atlantic coasts where this species is invasive (Krapp et al. 2006; Martínez and Adarraga 2008; Guerra-García et al. 2011; Minchin et al. 2012; Prato et al. 2013; Ros et al. 2013a, 2015, 2021; Cabezas et al. 2014; Molina et al. 2017). The species exhibits aggressive behavior among individuals, especially males in the presence of receptive females, which also may display parental care (Lim and Alexander 1986; Schultz and Alexander 2001).

##### Paracaprella aff. pusilla

Taxon classificationAnimaliaAmphipodaCaprellidae

55829437-9EFF-5B97-8AC3-B8C7CC74E9BA

Fig. 7

###### Material examined.

Uruguay • 6 males; La Paloma; 34°39'49.9"S, 54°10'10.7"W; 2–4 m deep, on filamentous algae; 7 June 2022. • 3 males, 8 females; La Paloma; 34°39'49.9"S, 54°10'10.7"W; 2–4 m deep, on filamentous algae; 1 September de 2022; Ramos T.; CCSEFC 343. • 1 male, 1 female; same collection data as for preceding; MNCN 20.04/20996.

**Figure 7.**
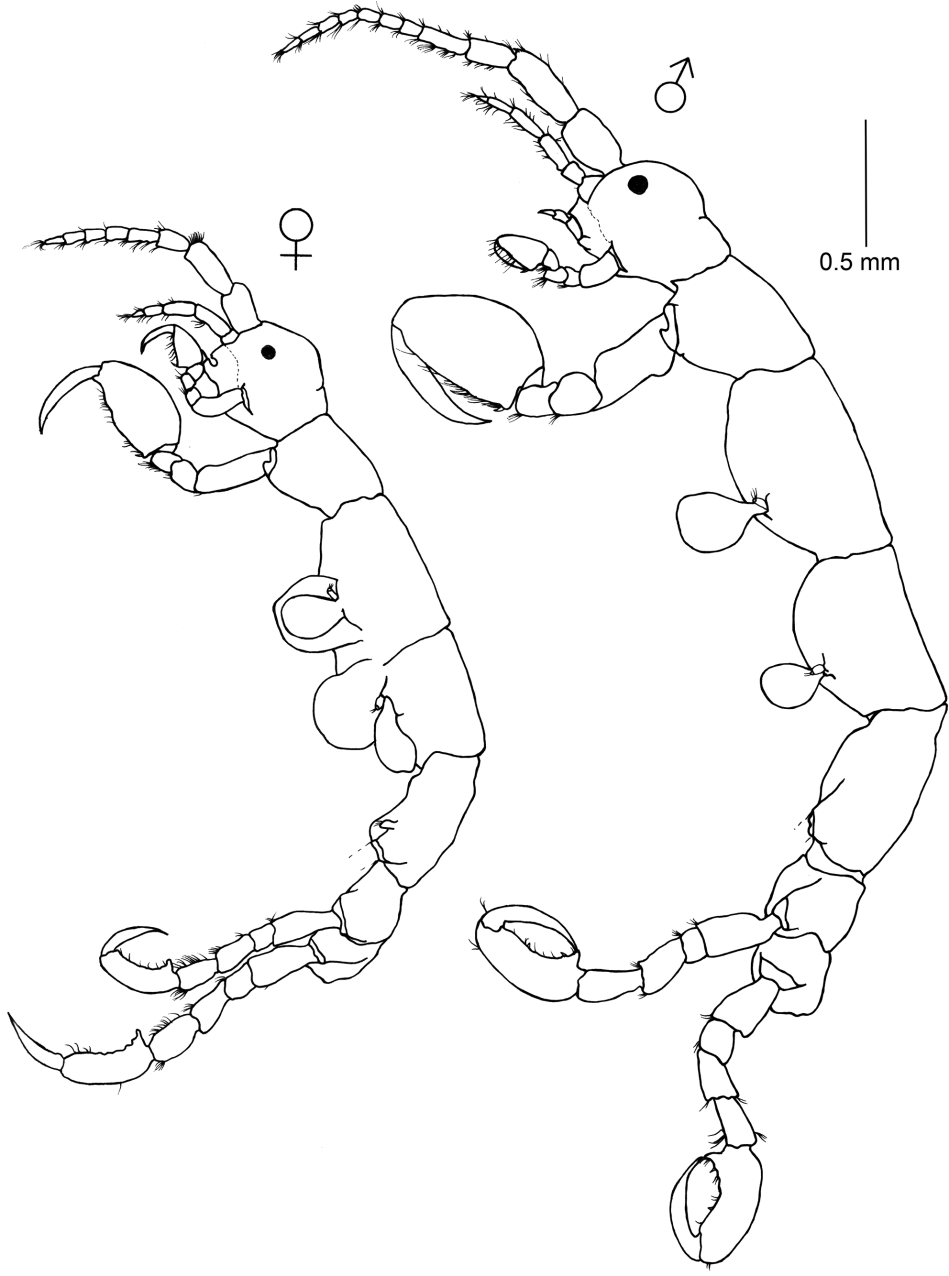
Paracaprella aff. pusilla. Lateral view of male and female collected from filamentous algae, rocky outcrops (2–4 m deep), 1 September de 2022, La Paloma (34°39'49.9"S, 54°10'10.7"W), Uruguay (MNCN 20.04/20996).

###### Remarks.

*Paracaprella pusilla* Mayer, 1890 is a tropical caprellid species recently introduced to the Eastern Atlantic coast of the Iberian Peninsula and the Mediterranean Sea (Ros and Guerra-García 2012; Ros et al. 2013b; Cabezas et al. 2019). The Atlantic coast of Central and South America has been postulated as the most likely native range for *P. pusilla* (Mayer 1903; McCain 1968; Carlton and Eldredge 2009; Cabezas et al. 2019). Indeed, *P. pusilla* is one of the dominant species in natural sheltered and artificial habitats of the coasts of southern Brazil (Ros et al. 2016), adjacent to the coast of Uruguay. Therefore, it was expected that the species is also distributed along the Uruguayan coast. The material collected during the present study morphologically resembles *P. pusilla*, mainly in the presence of the anterolateral projection of pereonite 2 and the proximal knob on the basis of gnathopod 2. However, the anterolateral projection of pereonite 2 is very small in the material from Uruguay, and Uruguayan specimens lack the small dorsal tubercle on pereonite 2, which is present in *P. pusilla* (Ros and Guerra-García 2012; Ros et al. 2013b). Gnathopod 2 and pleura of pereonites 3 and 4 also differ. Specimens from Uruguay are smaller than specimens of *P. pusilla* from other areas of the world. As specimens examined in the present study were mainly subadults, the morphological differences could be attributed to ontogenetic development. But we cannot exclude that the material of *Paracaprella* from this study could belong to an undescribed species, close to (but smaller than) *P. pusilla*. Collection of additional fresh material and further molecular and morphological analyses are encouraged to clarify the taxonomical status of *Paracaprella* in Uruguay.

### Key to species

**Figure 8.**
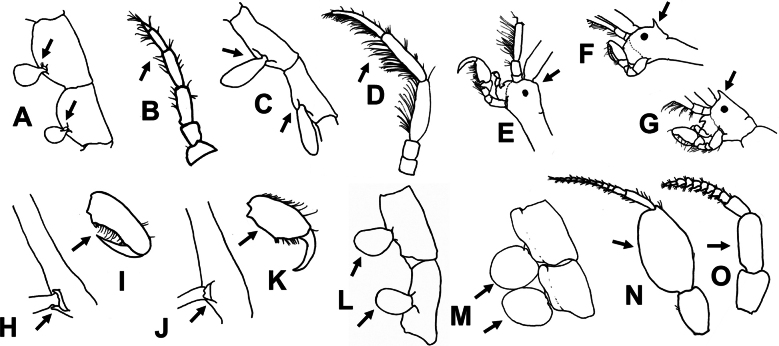
Details of the main morphological characters to identify the caprellids from Uruguay.

The following key for the seven caprellid species occurring in Uruguay is based on adult males and characters that do not need dissection of mouthparts (Fig. 8). A full redescription of *C. bathytatos* based on material from Uruguay can be found in Verdi and Celentano (2008).

**Table:** 

1	Pereopods 3 and 4 present (Fig. 8A). Antenna 2 scarcely setose (Fig. 8B)	**Paracaprella aff. pusilla**
–	Pereopods 3 and 4 absent (Fig. 8C). Antenna 2 setose (Fig. 8D)	**2**
2	Head without dorsal projection or acute rostrum (Fig. 8E)	**3**
–	Head with dorsal projection (Fig. 8F) or acute rostrum (Fig. 8G)	**4**
3	Ventral projection in pereonite 2 between gnathopods 2 present (Fig. 8H). Propodus palm of pereopods 5–7 concave (Fig. 8I)	***Caprella equilibra* Say, 1818**
–	Ventral projection in pereonite 2 between gnathopods 2 absent (Fig. 8J). Propodus palm of pereopods 5–7 convex (Fig. 8K)	***Caprella bathytatos* Martin & Pettit, 1998**
4	Head with dorsal projection (Fig. 8F)	***Caprella scaura* Templeton, 1836**
–	Head with rostrum (Fig. 8G)	**5**
5	Gills elongated (Fig. 8L)	**Caprella aff. penantis**
–	Gills rounded (Fig. 8M)	**6**
6	Antenna 1 with a globose article 2 (Fig. 8N). Propodus palm of pereopods 5–7 convex (Fig. 8K)	***Caprella andreae* Mayer, 1890**
–	Antenna 1 without globose article 2 (Fig. 8O). Propodus palm of pereopods 5–7 concave (Fig. 8I)	***Caprella dilatata* Krøyer, 1843**

## Discussion

This contribution represents the first comprehensive study of the caprellids from Uruguay, including six species from two genera, with the genus *Paracaprella* having its first record in Uruguayan waters. Of the species found, four are new records for the country, namely *Caprella andreae*, *C. dilatata*, *C. scaura*, and Paracaprella aff. pusilla, increasing the number of species found on the Uruguayan coast from three to seven. Despite the increase of more than 50% in the species recorded for Uruguay, the coastline of this country is still relatively less diverse compared to the coastlines of neighboring countries. Brazil, for example, hosts 26 cited species distributed across 12 genera, with greater diversity in the southeast and south coasts of the country (Mauro and Serejo 2015; Serejo and Siqueira 2018). However, we must consider the longer coastline of Brazil in comparison with Uruguay and its higher number of available marine habitats. Argentina presents 13 species in ten genera distributed along its entire coastline (López Gappa et al. 2006; Chiesa and Alonso 2014). A latitudinal pattern has been documented for caprellids where diversity and abundance are higher in temperate regions and tend to decrease towards the tropics and higher latitudes, where a limiting factor for diversity is the temperature of the surface water (Thiel et al. 2003; Guerra-García 2003b; Guerra-García et al. 2010b; Chunga-Llauce et al. 2023a). This pattern can also be observed in Brazil, with 68% of the cited species being found in the Warm Temperate Southwestern Atlantic (Spalding et al. 2007) and 32% in tropical zones (Mauro and Serejo 2015). The low number of species found in Uruguay may be related to the complexity of the highly variable environmental characteristics of the Uruguayan coast, mainly due to the interaction between freshwater from the Río de la Plata and seawater from the Atlantic Ocean. This constitutes a fluvial-marine system, resulting in significant temporal variations in water salinity, temperature, turbidity, and oxygen concentration, playing a central role in the structure of its communities (Gimenez 2006; FREPLATA 2004). Additionally, Uruguay is located at a crucial transition point in global ocean circulation, strongly influenced by the convergence in the Atlantic Ocean of subtropical and subantarctic water masses transported by the Brazil and Malvinas currents (Burone et al. 2021). The marine fauna of this region (the Uruguay-Buenos Aires shelf sensu Spalding et al. 2007) has a mixed origin, with a predominance of species from the subtropical region (Balech and Ehrlich 2008).

As mentioned, the studied region is a complex environment where, due to its characteristics, it is usually considered a coastline with low diversity. This is observed in planktonic species, which have a lower diversity pattern than expected, deviating from the usual pattern of higher diversity at lower latitudes (Boltovskoy et al. 1999; Woodd-Walker et al. 2002). Regarding the macrobenthic community, the discharge of the Río de la Plata has been considered as the main factor determining variations in distribution and composition patterns of decapods and molluscs (Maytia and Scarabino 1979), except for bivalves, which are not limited by salinity (Scarabino et al. 2015). The ecological distribution of other crustacean groups, such as amphipods and isopods, and polychaetes remains unknown (Calliari et al. 2003). Even though salinity plays a fundamental role in the variation in community structure, generally with lower species richness associated to lower salinities, its effects are very complex, multifactorial, and depend directly on the biology of the animals (Yamada et al. 2007). Although caprellids such as *C. equilibra*, *C. dilatata*, *and C. penantis* can be considered euryhaline (Takeuchi et al. 2003), the role of salinity and temperature has been considered critical to modulate interaction between *C. scaura* and *C. equilibra*, limiting, for example, the invasion success of *C. scaura* on the Iberian North-Atlantic coast. Indeed, the decreased seawater salinity and/or oxygen levels can weaken the ecophysiological resistance of *C. equilibra*, increasing its vulnerability to be replaced by *C. scaura* (Ros et al. 2021). Global warming can exacerbate interspecific interactions between caprellid species suggesting that climate change and ocean acidification can modify species distribution, community structure, and diversity (Lim and Harley 2018; Parretti et al. 2021).

The South American Atlantic coast has a peak diversity of macroalgae between latitudes 47°S and 54°S, with a significant decrease between 32°S and 37°S (which includes Uruguayan coasts), and an increase again towards lower latitudes. The discharge of the Río de la Plata could be the main abiotic factor responsible for this decrease in macroalgal diversity in Uruguay (Steigleder et al. 2019). The availability of habitats may be an essential factor driving the species richness of caprellids due to their epibiotic nature using substrata as a refuge (Lacerda and Masunari 2011; Ros et al. 2013a). As macroalgae are among the most common substrata for caprellids (Guerra García et al. 2010c), a decrease in algal diversity could also be involved in a lower caprellid diversity in Uruguay.

Therefore, the low diversity of caprellids in Uruguay must be considered in the context of variability of salinity and temperature due to river influence, and other potential factors, such as exposure to waves, availability of microhabitats, predation, and physicochemical conditions (Takeuchi et al. 2003). Future ecological studies will help to understand the ultimate biotic and abiotic factors responsible for the low diversity. Although our findings suggest that a combination of biotic and abiotic factors could be determinant in caprellid species richness, the taxonomic impediment (no specialists and few collections/material from the area) could also cause an underestimation of caprellid diversity. Further research is needed in different habitats (e.g., sediments) and depths, since caprellids have a wide distribution from the intertidal zone to depths over 7000 meters (Takeuchi et al. 2016). In addition, molecular analyses are needed to disentangle the taxonomical status of species such as Paracaprella aff. pusilla and Caprella aff. penantis. Despite the reduced number of species found, the present study marks the beginning of a better understanding of caprellid distribution and diversity on the Uruguayan coast.

## Supplementary Material

XML Treatment for Caprella andreae

XML Treatment for Caprella dilatata

XML Treatment for Caprella equilibra

XML Treatment for Caprella aff. penantis

XML Treatment for Caprella scaura

XML Treatment for Paracaprella aff. pusilla

## Footnotes

A detailed list of synonyms for this species can be consulted in McCain and Steinberg 1970.

A detailed list of synonyms for this species can be consulted in McCain and Steinberg 1970.

## Additional information

### Artificial Intelligence (AI) use

The authors accept full responsibility for the content of the manuscript, including the disclosure of any use of AI.

No AI tools were used in the preparation of this manuscript.

### Author ORCIDs

A. Verdi https://orcid.org/0000-0002-2084-6834

J.M. Guerra-García https://orcid.org/0000-0001-6050-4997

### Data availability

All of the data that support the findings of this study are available in the main text.
